# Corona bei Kindern: Die Co-Ki Studie: Relevanz von SARS-CoV-2 in der ambulanten pädiatrischen Versorgung in Deutschland

**DOI:** 10.1007/s00112-020-01050-3

**Published:** 2020-11-03

**Authors:** Silke Schwarz, Ekkehart Jenetzky, Hanno Krafft, Tobias Maurer, Christian Steuber, Till Reckert, Thomas Fischbach, David Martin

**Affiliations:** 1 Lehrstuhl für Medizintheorie, Integrative und Anthroposophische Medizin, Universität Witten/Herdecke, Alfred-Herrhausen-Straße 50, 58448 Witten, Deutschland; 2 Kinder- und Jugendpsychiatrie und -psychotherapie, Unimedizin Mainz, Mainz, Deutschland; 3 Praxis für Kinder und Jugendliche, ARCHE, Freiburg, Deutschland; 4 Berufsverband der Kinder- und Jugendärzte, Köln, Deutschland; 5 Universitätsklinik für Kinder- und Jugendmedizin, Universität Tübingen, Tübingen, Deutschland

**Keywords:** COVID-19, Pandemie, Kinder- und Jugendärzte, Versorgungsforschung, Epidemiologie, COVID-19, Pandemic, Family medicine, Public health, Epidemiology

## Abstract

**Hintergrund:**

In Deutschland werden über 80 % der Kinder und Jugendlichen von niedergelassenen Kinder- und Jugendärztinnen und -ärzten (KJÄ) betreut. Diese haben eine spezifische Perspektive auf die COVID-19-Pandemie.

**Methode:**

Zentrale Onlineerfassung von Fallzahlen, individuellen Fallbeschreibungen und Beobachtungen zu Infektion und Erkrankung mit SARS-CoV‑2 (www.co-ki.de).

**Ergebnisse:**

An der Fallzahlerfassung beteiligten sich bisher 557 KJÄ. Diese betreuen ca. 670.000 Kinder. Sie meldeten 9803 Kinder, die als Verdachtsfälle vorgestellt wurden. Die KJÄ selber hatten einen klinischen Verdacht auf SARS-CoV-2-Infektion bei 3654 Kindern. Bei 7707 Kindern wurden PCR-Testungen mittels Nasen‑/Rachenabstrich durchgeführt, von denen 198 Abstriche (2,6 %) positiv ausfielen. Zudem wurden 731 Kinder auf SARS-CoV-2-Antikörper getestet, mit einem Nachweis in 82 Fällen (11,2 %). Trotz initial positivem PCR-Test hatten 47 Kinder mindestens 2 Wochen danach einen negativen Antikörpertest. Die Abfrage nach Ansteckung eines Erwachsenen durch ein Kind ergab nur einen einzigen mutmaßlichen, nach telefonischer Rückfrage unwahrscheinlichen, Verdachtsfall.

**Diskussion:**

Aus ambulant-pädiatrischer Sicht sind COVID-19-Erkrankungen bei Kindern sehr selten. In unserem Kollektiv fand sich kein überzeugender Hinweis, dass Kinder eine relevante Infektionsquelle für SARS-CoV‑2 darstellen oder dass Kinder relevant gefährdet wären.

## Abstract (en)

**Background:**

In Germany over 80% of children and adolescents are in the ambulatory care of registered pediatricians. These have a specific perspective on the COVID-19 pandemic.

**Methods:**

For this reason, this professional group initiated a central recording of case numbers, individual case descriptions and observations on infections and illnesses with SARS-CoV‑2 (www.co-ki.de).

**Results:**

So far 557 pediatricians have participated. Together they care for ca. 670,000 children. They reported 9803 children who presented as suspected cases. The pediatricians themselves had a clinical suspicion of SARS-CoV‑2 infections in 3654 children. In 7707 children PCR tests were carried out using nose/throat swabs of which 198 (2.6%) were positive. In addition, 731 children were tested for SARS-CoV‑2 antibodies with detection in 82 cases (11.2%). Despite initially positive PCR tests, 47 children had a negative antibody test at least 2 weeks later. Our query as to infections of adults by children yielded only one case, which a telephone enquiry revealed as unlikely.

**Discussion:**

From an outpatient pediatric perspective COVID-19 is rare. There was no convincing evidence that children are a relevant source of infection for SARS-CoV‑2 nor that they are relevantly at risk.

## Hintergrund

Die aktuelle Studienlage deutet darauf hin, dass Kinder und Jugendliche eine geringere Rate symptomatischer SARS-CoV-2-Infektionen (COVID-19) aufweisen als Erwachsene und mehrheitlich keine oder nur milde Symptome entwickeln [1–3]. Zu schweren Verläufen kommt es bei Kindern selten, und in diesen Fällen handelt es sich gehäuft um Kinder mit Vorerkrankungen und Beeinträchtigungen des Immunsystems [4, 5]. Gemäß dem Lagebericht des Robert Koch-Institutes (RKI) vom 01.09.2020 gab es in Deutschland insgesamt 243.599 gemeldete Infektionen, von denen 8617 (3,5 % der Gemeldeten) unter 10 Jahre alt waren, und 16.193 (6,7 % der Gemeldeten) zwischen 10 und 19 Jahre alt waren [6]. Diese beiden Altersgruppen repräsentieren jedoch jeweils 9,2 % der Bevölkerung. Deshalb stellen sich die Fragen, ob nicht alle Kinder erfasst wurden, die eine Infektion durchgemacht haben [7], oder ob Kinder und Jugendliche im Verhältnis seltener durch eine Infektion betroffen sind. Darüber hinaus ist leider nicht bekannt, wie viele der vom RKI erfassten Kinder symptomatisch waren, und ob ihre etwaigen Symptome auf SARS-CoV‑2 zurückzuführen sind. Todesfälle durch COVID-19 sind bei Kindern und Jugendlichen extrem selten. Bis zum 01.09.2020 wurden deutschlandweit 3 Todesfälle mit möglichem Zusammenhang mit SARS-CoV‑2 bei Menschen zwischen 0 und 19 Jahren festgestellt. Diese hatten jeweils Vorerkrankungen [6]. Ob Kinder grundsätzlich eine geringere Infektionsprävalenz für eine SARS-CoV-2-Infektion haben, ist aufgrund fehlender Referenztestungen unklar [8–11]. Häufig werden Kinder- und Jugendärztinnen und -ärzte (KJÄ) mit den Konsequenzen der eingreifenden Maßnahmen, wie z. B. Kindergarten- und Schulschließungen, und deren sozialpädiatrischen Folgen konfrontiert. Weiterhin stellen sie sich die Frage nach der gesundheitlichen Relevanz der SARS-CoV-2-Infektion und COVID-19-Erkrankung im Kindes- und Jugendalter. Die vorliegende Erhebung stellt die Pandemiesituation bei Kindern aus Sicht der ambulant-pädiatrischen Regelversorgung dar: Sie bietet eine ambulante Ergänzung zu der laufenden Untersuchung zur stationären Behandlung der Deutschen Gesellschaft für Pädiatrische Infektiologie (DGPI) dar und erfolgte in Absprache mit dieser [4].

## Methode

In Deutschland werden über 80 % der Kinder und Jugendlichen von niedergelassenen KJÄ betreut. Diese Berufsgruppe hat eine fortlaufende Onlineerfassung von Fallzahlen, individuellen Fallbeschreibungen und Fachmeinungen zu der Infektion mit SARS-CoV‑2 und der COVID-19-Erkrankung initiiert. Ein positives Ethikvotum der Universität Witten/Herdecke liegt dazu vor. Für die Erfassung wurden alle im Berufsverband der Kinder- und Jugendärzte (BVKJ) aktiven und per E‑Mail erreichbaren KJÄ (*n* = 5600) in Deutschland am 08.05.2020 und 15.06.2020 durch den Präsidenten des BVKJ und den Letztautor per E‑Mail zur Teilnahme am Online-Survey www.co-ki.de eingeladen. Die Aufforderung zur Teilnahme erfolgte explizit auch für die Situation, dass keine Fälle von COVID-19- oder SARS-CoV-2-Positivität in der eigenen Praxis vorliegen. Diese Publikation stellt eine deskriptive Zwischenauswertung dar. Der verwendete Fragebogen ist in Tab. 1 dargestellt.

**Table 1:** 

Alle Fragen beziehen sich auf Ihre Zahlen seit Ihrer letzten Eingabe
1. Bitte tragen Sie hier die ersten drei Ziffern der Postleitzahl Ihres Praxisortes ein
2. Wie viele Kinder mit V. a. SARS-CoV-2-Infektion (Verdacht durch Eltern oder Kontakt mit SARS-CoV-2-Infizierten) wurden in Ihrer eigenen Sprechstunde vorgestellt?
3. Bei wie vielen dieser Kinder hatten Sie ebenfalls den Verdacht auf eine SARS-CoV-2-Infektion?
4. Wie viele Kinder wurden in Ihrer Sprechstunde auf SARS-CoV-2-Viren (PCR, Nasen‑/Rachenabstrich) getestet?
5. Wie viele dieser Kinder hatten ein positives Testergebnis (PCR, Nasen‑/Rachenabstrich)?
6. Wie viele Kinder in Ihrer Sprechstunde wurden auf SARS-CoV-2-Antikörper (Blut/Serum) getestet?
7. Wenn Sie es wissen, können Sie hier eintragen, welche Tests (Labor oder Schnelltest, Hersteller) verwendet wurden
8. Bei wie vielen dieser Kinder gab es ein positives Testergebnis mit SARS-CoV-2-Antikörpern (Blut/Serum)?
9. Wie viele Kinder hatten Sie, die PCR (Nasen/Rachen-Abstrich) positiv, aber mindestens 2 Wochen danach AK (Blut/Serum) negativ waren?
10. Wie viele der positiv (PCR oder AK) Getesteten hatten zu irgendeinem Zeitpunkt Symptome?
11. Wie viele der positiv Getesteten (PCR/AK) hatten keine Symptome?
12. Wie viele Kinder mit SARS-CoV-2-Infektionen mussten ins Krankenhaus?
13. Wir freuen uns über eine detaillierte Schilderung Ihrer medizinischen Beobachtungen. Insbesondere interessiert uns die Frage, welche Kinder andere Menschen anstecken und welche nicht. Auch Ihre Gedanken und Wahrnehmungen der Gesamtsituation, und was es für die Kinder bedeutet, interessiert uns. Kontaktieren Sie uns gerne, wenn Sie besonders interessante Fälle zu besprechen haben

## Ergebnisse

**Figure 1:**
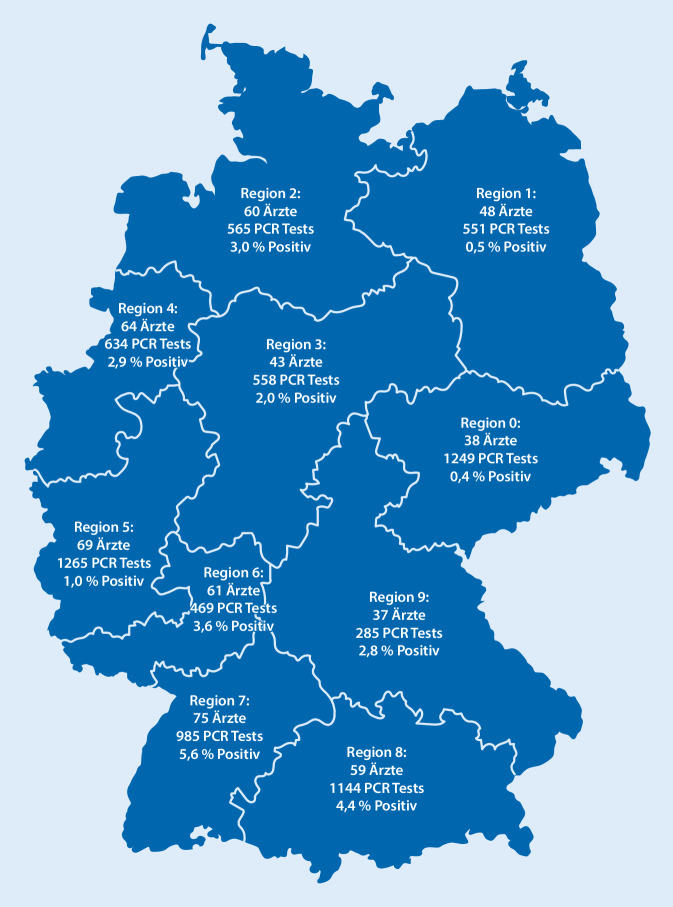

An der Fallzahlerfassung beteiligten sich bisher 557 KJÄ (Stand 01.09.2020). Diese KJÄ meldeten insgesamt 9803 Kinder und Jugendliche, die aufgrund eines Verdachts der Eltern oder eines Kontaktes mit einem SARS-CoV-2-Infizierten in der Sprechstunde vorgestellt wurden. Die KJÄ ihrerseits hatten den klinischen Verdacht auf eine SARS-CoV-2-Infektion bei 3654 Kindern, wobei einzelne KJÄ mehr eigene Verdachtsfälle als Vorstellungen mit Verdacht meldeten, und das Wort „ebenfalls“ in Frage 4 wohl ignorierten, weshalb die 3654 nicht eine reine Teilmenge von den 9803 sind. Bei 7707 Kindern und Jugendlichen wurden durch 443 KJÄ PCR-Testungen mittels Nasen‑/Rachenabstrichen durchgeführt. Es wurden mehr Kinder getestet als die, bei denen ein Verdachtsgrund bestand (Wunsch der Eltern, amtliche Anordnung etc.). Davon waren 198 Abstriche (2,6 %) bei 93 Ärzten positiv. Die berichtenden KJÄ führten an, dass in vielen Fällen trotz Infektionsverdacht aus verschiedenen Gründen nicht getestet wurde: z. B. fehlendes Testmaterial oder Einschätzung fehlender Notwendigkeit, da bei mildem Verlauf und positiv getestetem Familienmitglied ohnehin eine Quarantäne angezeigt war. Kinder- und Jugendärztinnen und -ärzte aus allen 10 Postleitzahlregionen nahmen teil. Der Anteil an positiven PCR-Tests zu der Gesamtzahl an PCR-Tests war höher im Südwesten und Nordwesten Deutschlands (Abb. 1).

**Figure 2:**
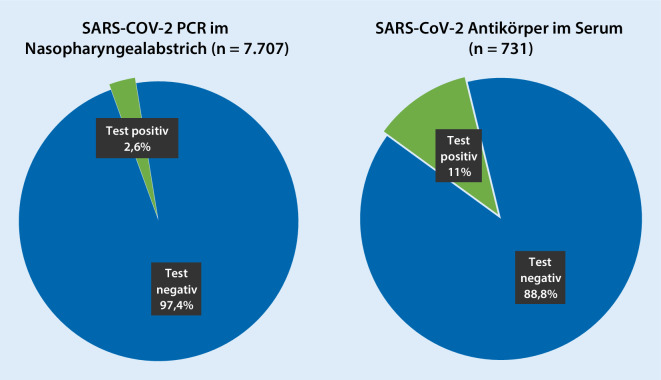

In 731 Fällen wurden durch 157 KJÄ eine Antikörpertestung durchgeführt, wovon 82 (11,2 %) positiv ausfielen (Abb. 2). Von den 140 KJÄ, die angegeben haben, welchen Test sie verwendeten, schickten 101 ihre Proben in Labore. Schnelltests wurden von 39 KJÄ genannt: entweder IgG alleine (Euroimmun (33 %), Diasorin (5 %), Microgen (2,5 %)), oder mit IgM (Roche (13 %), Cleartest (5 %), Nadal (2,5 %)) oder mit IgA (Euroimmun 10 %). Trotz initial positivem PCR-Test hatten 47 Kinder mindestens 2 Wochen danach einen negativen Antikörpertest. Neunundzwanzig Kinder mit V. a. COVID-19 wurden stationär aufgenommen. Allerdings lag bei 16 dieser 29 Fälle kein positiver Virusnachweis vor. Im Rahmen der Individualfallerfassung wurde bei 5 Fällen, die stationär aufgenommen wurden, ein dem Kawasaki-Syndrom ähnliches Krankheitsbild dokumentiert. Diese 5 Fälle hatten einen negativen Nasen‑/Rachenabstrich für SARS-CoV‑2, waren also möglicherweise keine COVID-19-Patienten. Weitere Informationen hierzu erfassten die Kliniken in der DGPI-Umfrage [7].

Die KJÄ wurden außerdem gebeten, mutmaßliche Ansteckungsketten qualitativ zu schildern: So wurden mehrere Beispiele von Großfamilien beschrieben, in denen ein Kind außerfamiliär angesteckt wurde und die Infektion trotz intensivem, körpernahem Kontakt innerhalb der Familie nicht weitergab. 99,8 % der KJÄ äußerten, keinen Fall gesehen zu haben, in dem ein Erwachsener durch ein Kind infiziert wurde. Es gab nur einen mutmaßlichen Verdachtsfall einer Übertragung der SARS-CoV-2-Infektion von einem Kind auf einen Erwachsenen. Dabei handelte es sich um ein 2‑jähriges Mädchen, welches einen Tag lang Fieber hatte und nicht getestet wurde. Es hatte in der Kita Kontakt zu einem Kind, dessen Familie einen fraglichen Kontakt mit COVID-19 hatte. Die Eltern hatten 5 Tage später leichte Hals- und Kopfschmerzen. Die Mutter wurde PCR-positiv getestet. Der Vater wurde nicht PCR-getestet, wurde aber später antikörpernegativ getestet. Wir konnten durch den eintragenden KJA die Mutter des oben genannten Verdachtspatienten (die Tochter des eingebenden KJA) telefonisch kontaktieren und erfuhren, dass bei dem fast 3‑jährigen Kind weder PCR- noch Antikörpertestungen durchgeführt wurden. Außerdem waren beide Eltern berufstätig und einkaufend unterwegs gewesen. Somit bleibt fraglich, ob sich die Mutter beim Kind angesteckt hat.

Zusätzlich zu den quantitativen Angaben gab es viele Spontanbeschreibungen über Familien, in denen eben keine Ansteckung von PCR-positiven Kindern auf Erwachsene stattfand. Im Folgenden sind einige Fallbeispiele aufgeführt; bei den ersten beiden Fällen haben sich die Kinder möglicherweise bei anderen Kindern angesteckt:

1. Junge, 7 Jahre alt. Vorstellungsgrund Husten. Nach 4 Tagen erneute Vorstellung wegen Fieber bei Belastung und persistierendem Husten. Testung wegen Verdachtsfall auf Coronainfektion in der Schulklasse. Ergebnis: PCR-positiv. Vollständige Genesung nach einer Woche. Die Abstriche, die vom Gesundheitsamt von den Eltern und Geschwistern entnommen wurden, sind verloren gegangen. Klinisch erkrankte niemand aus der Familie.
2. Mädchen, 10 Monate alt. Vorstellungsgrund: Fieber, Abgeschlagenheit, Weinerlichkeit, Trinkverweigerung. Fünf Tage vor Erkrankungsbeginn von fremdem Kind angehustet worden. PCR-positiv. Stationärer Aufenthalt mit Infusionstherapie. Entlassung nach 5 Tagen mit hohem Fieber. Anschließend morbiliformer Hautausschlag, fast am gesamten Integument (Arme, Beine, Rumpf), nach 12 Tagen abblassend. Weder Schwester (Schnuller ausgetauscht) noch Eltern angesteckt.
3. Kind, 1 Jahr alt. Hohes Fieber, PCR-positiv. Keine der 10 Kontaktpersonen positiv getestet, auch nicht die Großeltern des Kindes, die besucht wurden.
4. Kind, 4 Jahre alt. Produktiver Husten, mäßiges Fieber, PCR-positiv. Hat weder Vater, Mutter noch die 1‑jährige Schwester angesteckt.
5. Junge, 4 Jahre alt. Erkältung ohne Fieber. PCR-positiv. Keine weiteren angesteckten Familienmitglieder. Zwei Geschwisterkinder, nächtliches Fieber und Husten für 1 Woche, nicht schwer erkrankt. Anamnestisch rezidivierende Bronchitiden. Nach 10 Tagen keine Symptome mehr. Eltern symptomlos.
6. Mädchen, 8 Jahre alt. PCR-positiv. Intensivmedizinische Betreuung bei schwerer Pneumonie. Außer Adipositas keine Risikofaktoren/Vorerkrankungen. Eltern und Bruder ohne Krankheitszeichen, kein Erregernachweis bei Mutter trotz engem Kontakt.
7. Kind mit respiratorischen Symptomen und Durchfall, hatte sich in der Schule angesteckt. PCR-positiv. Die 5‑köpfige Familie ist mehrfach getestet worden und war negativ.
8. Mädchen, 11 Jahre alt. Erhöhte Temperaturen und leichte Kopfschmerzen. Vom Vater angesteckt, der sich wiederum bei einem italienischen Kollegen angesteckt hatte. Vater und Mädchen PRC-positiv. Das Mädchen kuschelt gerne mit Mutter, Stiefvater und einjährigem Bruder, hat dennoch keinen von ihnen angesteckt.

## Diskussion

Diese Versorgungsstudie stellt die Versorgungsrealität von 557 niedergelassenen KJÄ in der gegenwärtigen Pandemie dar. Bei durchschnittlich 1200 Kindern und Jugendlichen pro Praxis betreuen diese 557 KJÄ ca. 0,67 Mio. Kinder und Jugendliche.

Die meldenden KJÄ hatten im Durchschnitt ca. 18 Verdachtsfälle, wobei berücksichtigt werden muss, dass viele KJÄ gar keinen Verdachtsfall in der Praxis hatten und deshalb auch keine Meldung durchführten.

Von den 7707 Nasen‑/Rachenabstrichen (PCR) waren 2,6 % und von 731 Antikörpertesten waren 11,2 % positiv. Die höhere Rate an Antikörperpositivität hängt mit der Tatsache zusammen, dass mehr Kinder getestet wurden, die zuvor Symptome und/oder einen positiven PCR-Test hatten. Die Tatsache, dass bei 47 Kindern trotz positivem PCR-Test nach mehr als 2 Wochen keine Antikörper nachgewiesen werden konnten, wirft die Frage auf, ob die Sensitivität der Antikörpertests zu gering war, oder ob diese Kinder (noch) keine oder zu wenig Antikörper bildeten. Aktuelle Untersuchungsergebnisse ergaben, dass es bis zur Antikörperbildung bis zu 19 Tage dauern kann [8]. Während IgG oft alleine getestet wurde, gaben keine KJÄ an, nur IgA oder IgM getestet zu haben, was angesichts der niedrigen, zeitabhängigen Sensitivität von IgA und IgM bei SARS-CoV‑2 durchaus sinnvoll erscheint.

Viele KJÄ äußern sich erstaunt über die niedrige Inzidenz von SARS-CoV‑2. Eine Herausforderung stellte in der Anfangszeit die fehlende Verfügbarkeit von Tests dar. Das spiegelt sich in der geringen Anzahl durchgeführter Tests bei symptomatischen Kindern wider. Das RKI gibt 0,18 % [6] laborbestätigte SARS-CoV-2-Infektionen bei Kindern und Jugendlichen an, kalkuliert auf dem Hintergrund einer Grundgesamtheit von 13,5 Mio. Kindern in Deutschland (Stand 01.09.2020). Insgesamt ist die Rate der nachweislich infizierten Kinder und Jugendlichen, in der vorliegenden Studie bislang, ähnlich derjenigen des RKI, niedrig.

Fünf der 29 stationären Fälle wurden im Zusammenhang mit den weltweit gemeldeten Kawasaki-ähnlichen Krankheitsverläufen bei Kindern gesehen. Auf diesem Hintergrund ist festzustellen, dass die durchschnittliche Kawasaki-Inzidenz in Deutschland 9/100.000 Kinder beträgt, und dass das Kawasaki-Syndrom auch vor der Pandemie mit anderen Coronaviren assoziiert war [12, 13]. Die Tatsache, dass alle 5 Fälle negativ für SARS-CoV‑2 in der PCR getestet wurden, schließt nicht aus, dass sie mit SARS-CoV‑2 assoziiert waren, da das Kawasaki-ähnliche Syndrom eine Späterscheinung zu sein scheint, also zu einem Zeitpunkt auftritt, bei dem die Antigene oft nicht mehr nachweisbar sind [12].

Auf die explizite Frage, ob Hinweise für Ansteckungen von Erwachsenen durch Kinder vorlagen, wurde nur ein mutmaßlicher Verdachtsfall gemeldet. Hingegen wurde spontan über viele Familien berichtet, in denen Kinder Mitmenschen trotz engen Kontaktes nicht ansteckten. Die Schilderung aus der ambulanten Pädiatrie, dass die meisten Kinder sich bei den Eltern angesteckt haben, deckt sich mit den Erhebungen des COVID-19-Krankenhausregisters der DGPI [4]. In unserem Kollektiv fand sich kein überzeugender Hinweis dafür, dass Kinder eine relevante Infektionsquelle darstellen. Bisherige Vermutungen zur Rolle von Kindern in der Dynamik der Pandemie sind aus Influenzapandemien übertragen worden. Sie können aufgrund der vorliegenden Daten dieser ambulanten und der stationären [4] Erfassung in Deutschland zur SARS-CoV-2-Pandemie nicht bestätigt werden. Die (erstaunlich) wenigen, bisher durchgeführten Untersuchungen zu diesem Thema zeigen eine sehr niedrige Ansteckungsrate durch Kindern [14]. Womöglich schützen Kinder ältere Menschen sogar vor einem schweren COVID-19-Verlauf [15].

Die aktuelle Pandemie mit SARS-CoV‑2 ist komplex, die unsichere Datenlage eine große Herausforderung. Diese erste Auswertung der ambulanten Pädiatrie weist naturgemäß Limitationen auf, da die Beteiligung zwar bei ca. 10 % liegt, sich aber ein „reporting bias“ hin zur Dokumentation positiver und/oder symptomatischer Fälle mutmaßen lässt. Dies liegt vermutlich daran, dass KJÄ ohne Fälle sich seltener an der Datenerhebung beteiligten.

Eine Vielzahl der SARS-CoV-2-Abstriche wurde nicht in Kinder- und Jugendarztpraxen, sondern in Gesundheitsämtern, Kliniken und durch andere ärztliche Fachrichtungen vorgenommen. Diese Studie stellt die direkte Wahrnehmung der niedergelassenen Pädiater dar. Daher können die Ergebnisse dieser Studie nicht ohne Weiteres verallgemeinert werden.

Die Autoren sind weiterhin sehr interessiert daran, Berichte von Ansteckungsketten durch Kinder hin zu Erwachsenen und umgekehrt zu erhalten. Im Hinblick auf Kindergarten- und Schulöffnungen und die damit einhergehenden, sich bisher eher nicht bestätigenden [16] Befürchtungen ist eine multiperspektivische Erfassung der Situation der Kinder von großer Bedeutung, weshalb dieses Register eine Ergänzung zum stationären Register und den Daten der Gesundheitsämter darstellt.

## Fazit für die Praxis

Weiterhin sind alle KJÄ zur Teilnahme an www.co-ki.de eingeladen, sowohl bei SARS-CoV-2-Verdacht als auch bei COVID-19-Erkrankung und bei fehlenden Fällen in der Praxis.

## Abkürzungen

COVID-19: „Corona virus disease 2019“
Co-Ki: Corona-bei-Kindern-Studie
PCR: Polymerase-Kettenreaktion
RKI: Robert Koch-Institut
SARS-CoV‑2: „Severe acute respiratory syndrome coronavirus 2“ (schweres akutes Atemwegssyndrom-Coronavirus 2)
